# Methods in causal inference. Part 2: Interaction, mediation, and time-varying treatments

**DOI:** 10.1017/ehs.2024.32

**Published:** 2024-10-01

**Authors:** Joseph A. Bulbulia

**Affiliations:** Victoria University of Wellington, Wellington, New Zealand

**Keywords:** DAGs, mediation, moderation, SWIGs, time-varying treatments

## Abstract

The analysis of ‘moderation’, ‘interaction’, ‘mediation’ and ‘longitudinal growth’ is widespread in the human sciences, yet subject to confusion. To clarify these concepts, it is essential to state causal estimands, which requires the specification of counterfactual contrasts for a target population on an appropriate scale. Once causal estimands are defined, we must consider their identification. I employ causal directed acyclic graphs and single world intervention graphs to elucidate identification workflows. I show that when multiple treatments exist, common methods for statistical inference, such as multi-level regressions and statistical structural equation models, cannot typically recover the causal quantities we seek. By properly framing and addressing causal questions of interaction, mediation, and time-varying treatments, we can expose the limitations of popular methods and guide researchers to a clearer understanding of the causal questions that animate our interests.

## Graphical abstract

**Figure:**
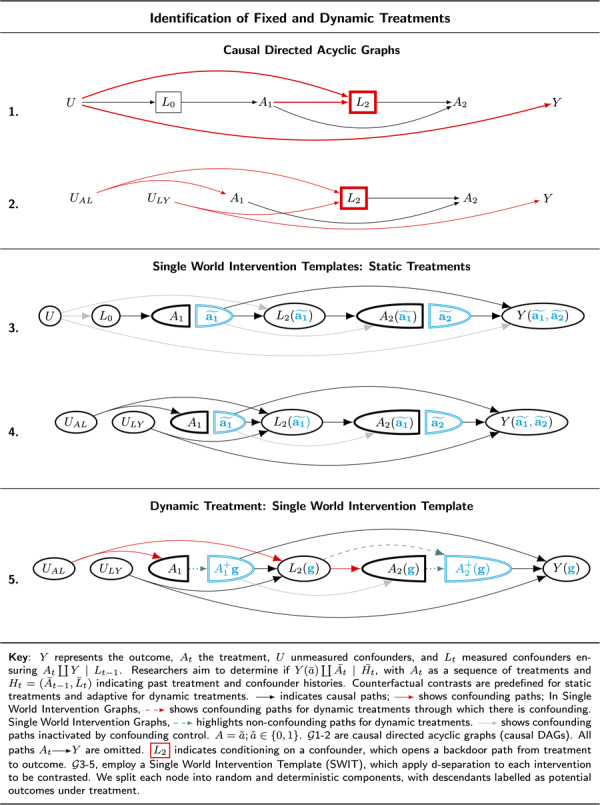

## Introduction

The young Charles Darwin was a keen fossil hunter and amateur geologist. In August 1831, he accompanied the geologist Adam Sedgwick to the Glyderau mountain range in northern Wales.

We spent many hours in Cwm Idwal, … but neither of us saw a trace of the wonderful glacial phenomena all around us; we did not notice the plainly scored rocks, the perched boulders, the lateral and terminal moraines. Yet these phenomena are so conspicuous that … a house burnt down by fire did not tell its story more plainly than did this valley. If it had still been filled by a glacier, the phenomena would have been less distinct than they now are. (Darwin, 1887: 25)

This ‘striking instance of how easy it is to overlook phenomena, however conspicuous’ (Darwin, 1887: 25) is cited in cultural evolution to emphasise the importance of theory for organising observations (Wilson, 2008). However, the importance of theory to scientific discovery carries broader relevance: it applies to the statistical methods scientists routinely apply to the data they collect. Without a clear framework that relates statistical models to observations, the understanding we seek from our data remains elusive.

Across many human sciences, we apply statistical models to data and report ‘moderation’, ‘interaction’, ‘mediation’ and ‘longitudinal growth’. How are we to interpret the results of these models? It is often unclear. The confidence with which investigators report findings does not make interpretation any clearer. The problem is that investigators are typically interested in causal questions. Answering a causal question requires a careful workflow that begins by defining the causal quantities and the target population of interest. We specify a causal quantity, or estimand, as the contrast between counterfactual outcomes under two (or more) clearly defined interventions. Mathematical proofs establish that we can consistently estimate the size of this counterfactual difference using data, provided certain assumptions are met (discussed below). The subsequent steps in a causal inferential workflow involve assessing the credibility of these necessary assumptions, constructing appropriate estimators, and obtaining relevant data. Only after these steps should statistical analysis be attempted. Without adhering to a causal inferential workflow, the associations derived from statistical models will reflect true causal relationships only by chance, regardless of the sophistication of our statistical methods (Westreich & Greenland, 2013).

There is good news. Advances in the health sciences, computer science and economics have fostered a common vocabulary and established robust workflows, enabling investigators to formulate causal questions that can be addressed with data. These developments facilitate the evaluation of the necessary assumptions for obtaining consistent estimates, the construction of valid estimators, and the application of statistical models at the culmination of the workflow. This conceptual framework, grounded in mathematical proofs, empowers investigators to effectively clarify, communicate, and evaluate causal questions using observational data. The consensus that has emerged in causal inference over the past several decades is, in my view, as transformative for the human sciences as the theory of glaciation was for geology or Darwin's theory of evolution was for biology. By reframing questions of interaction, mediation and time-varying treatments as causal inquiries, I aim to clarify the framework's interest, relevance and power.

Several excellent resources clarify workflows for causal inference, guiding researchers from stating causal questions to communicating results (Hernán & Robins, 2024; Laan et al., 2023; Montgomery et al., 2018; Morgan & Winship, 2014; Neal, 2020; Pearl, 2009; Tibshirani et al., 2024; T. J. VanderWeele, 2015).

Here, my ambition is focussed.

Part 1 considers how to formulate causal questions when our interest lies in comparing effect magnitudes between groups (effect modification).

Part 2 considers how to formulate causal questions to assess the joint effects of two independent interventions (interaction).

Part 3 considers how to formulate causal questions to assess the joint effects of two dependent interventions (mediation analysis).

Part 4 considers how to formulate causal questions when evaluating two or more sequential treatments of the same kind (time-varying treatments).

I begin with a brief introduction to key concepts and terminology.

### Fundamental assumptions for causal inference

Consider indicators *A* and *Y* measuring states of the world. Let *A* denote the ‘treatment’ or ‘exposure’ and *Y* the ‘outcome’. For unit *i*, we say that *A*_*i*_ causes *Y*_*i*_ if changing *A*_*i*_ from one level, say *A*_*i*_ = a*, to another level, *A*_*i*_ = *a*, leads to a different outcome for *Y*_*i*_. We assume that *A*_*i*_ occurs before *Y*_*i*_. To compare these outcomes, we use the notation 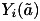, which represents the outcome for unit *i* under the treatment level 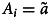. To determine whether 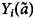 quantitatively differs under two treatment levels on the difference scale, we would compute the contrast *Y*_*i*_(a*) − *Y*_*i*_(*a*). If *Y*_*i*_(a*) − *Y*_*i*_(*a*) ≠ 0, we would say there is a causal effect of *A* on *Y* for individual *i*.

Note that, for any given treatment of *A* for unit *i*, we can only observe one level of treatment. Therefore, we refer to *Y*_*i*_(a*) − *Y*_*i*_(*a*) ≠ 0 as a counterfactual contrast, or equivalently, as a contrast of potential outcomes. Because an individual may only receive one of two treatments at any given time, individual causal effects cannot generally be observed. However, when certain assumptions are satisfied, we may compute average treatment effects by aggregating individual observations under different treatment conditions. For a binary treatment, the difference in the average of the potential outcomes under two different treatment levels for the population from which a sample is drawn may be expressed as the difference in mean outcomes: 𝔼[*Y*(1)] − 𝔼[*Y*(0)] or equivalently as the average of the differences of the potential outcomes: 𝔼[*Y*(1) − *Y*(0)].

This counterfactual contrast represents the quantity obtained from an ideally conducted randomised controlled trial, where any common cause of the treatment and outcome would occur only by chance. Three fundamental assumptions for computing average treatment effects. Although these are typically satisfied in well-conducted randomised controlled trial, they may not be hold for observational or ‘real world’ data:

1. *Causal consistency* –The treatment level received by each unit matches the treatment defined by the protocol, remaining consistent within the treatment arms to be compared. There must be at least two arms for comparison.
2. *(Conditional) exchangeability* – Potential confounders that might affect the outcomes under different treatment conditions are balanced across all arms. In randomised controlled trials this balance is generally achieved by randomisation. In observational studies it must be addressed using methods of covariate adjustment.
3. *Positivity (or Overlap)* – For every combination of covariates in the target population, there is a non-zero probability of receiving each treatment condition. In other words, every unit with a given set of covariates must have some chance of being observed in all treatment arms being compared. This ensures that all subgroups in the population have exposure to each treatment condition, enabling comparisons across those subgroups. Violations of positivity occur when certain subgroups have no overlap in treatment conditions (e.g., a covariate perfectly predicts treatment assignment), which can lead to biased or unstable estimates of causal effects (refer to Westreich & Cole, 2010; Bulbulia et al., 2023).

Note that real-world experiments may fail to meet these assumptions (Bulbulia, 2024c; Hernán & Robins, 2017). Practical limitations, such as imperfect randomisation, non-compliance, or measurement error, can lead to violations of consistency, exchangeability, or positivity. Our interest here is specifically restricted to causal inference using observational or ‘real world’ data, where these assumptions are often particularly challenging to satisfy.

### Schematic workflow for inferring causal effects from real-world data before stating statistical estimators and performing statistical analysis

In causal inference, we do not apply statistical models to data until after we have stated a causal question and considered whether and how the question may be identified from the data. We take the following steps *before* considering a statistical estimator or estimation:

1. *State a well-defined treatment* – clearly define the treatment (or equivalently exposure) that states the hypothetical intervention to which all population members will be exposed. For example, the treatment ‘weight loss’ is a vaguely stated intervention because there are many ways one can lose weight – exercise, diet, depression, cancer, amputation and others (Hernán & Robins, 2024). The intervention ‘weight loss by at least 30 minutes of vigorous exercise each data’ is more clearly defined (Hernán et al., 2008).
2. *State a well-defined outcome* – specify the outcome measure so that a causal contrast is interpretable. For example, ‘well-being’ is arguably a vaguely stated outcome. However, the outcome, ‘psychological distress measured one year after the intervention using the Kessler-6 distress scale’ (Kessler et al., 2002) is more clearly defined.
3. *Clarify the target population* – define the population to whom the results will generalise. The eligibility criteria for a study will define the source population from which units in the study are sampled. However, sampling from the source population may yield a study population that differs from the source population in variables that modify the effects of treatment (Bulbulia, 2024b; Dahabreh et al., 2019; Dahabreh & Hernán, 2019; Stuart et al., 2018). Investigators may also seek to generalise beyond the source population, which requires additional assumptions and may require additional knowledge (Bareinboim & Pearl, 2013; Dahabreh & Hernán, 2019; Deffner et al., 2022; Pearl & Bareinboim, 2022; Westreich et al., 2017).
4. *Evaluate whether treatment groups, conditional on measured covariates, are exchangeable* – the potential outcomes must be independent of treatments conditional on measured covariates (Angrist & Pischke, 2009; Hernán & Robins, 2024; Morgan & Winship, 2014; Neal, 2020).
5. *Ensure treatments to be compared satisfy causal consistency* – the versions of treatments over which a causal effect is estimated must be independent of the potential outcomes to be compared conditional on measured covariates (Hernán & Robins, 2024; T. J. VanderWeele & Hernán, 2012, 2013).
6. *Check if the positivity assumption is satisfied* – there must be a non-zero probability of receiving each treatment level at every level of covariate required to satisfy the conditional exchangeability assumption, and if there are many versions of treatment, to satisfy the causal consistency assumption (Westreich & Cole, 2010).
7. *Ensure that the measures relate to the scientific questions at hand* – in particular, evaluate structural features of measurement error bias (Bulbulia, 2024b; Hernán & Robins, 2024; T. J. VanderWeele & Hernán, 2012).
8. *Consider strategies to ensure the study group measured at the end of the study represents the target population* – if the study population differs in the distribution of variables that modify the effect of a treatment on the outcome at both the beginning and end of treatment, the study will be biased when there is a treatment effect; as such, investigators must develop strategies to address attrition, non-response and structural sources of bias from measurement error (Bulbulia, 2024b; Hernán et al., 2004; Hernán & Robins, 2017; Hernán, 2017).
9. *Clearly communicate the reasoning, evidence, and decision-making that inform steps 1–8* – provide transparent and thorough documentation of the decisions in steps 1–8. This includes detailing investigators’ causal assumptions and any disagreements about these assumptions (Ogburn & Shpitser, 2021).

### Conventions used in this article

Table 1 reports our variables. Table 2 describes our graphical conventions. Here, we use two types of graphical tools to clarify causal questions: causal directed acyclic graphs and single world intervention graphs. (Refer to supplementary materials S1 for a glossary for commonly used causal inference terms.)

**Table 1.** Terminology

Definitions of Symbols	
Symbol	Description
*X*	A capital letter representing a random variable.
*X* = *x*	A small letter indicating the random variable *X* fixed at value *x*.
*A*	The treatment or, equivalently, the exposure.
*A* = *a*	Treatment *A* fixed to level *a*.
*Y*	The outcome variable.
*Y*(*a*)	The potential or counterfactual outcome when *A* = *a*. Represented also as *Y*^*a*^ or *Y*_*a*_.
*L*	Measured confounder(s): typically comprises a set of variables.
*U*	Unmeasured confounder.
*F*	Effect-modifier (or ‘moderator’) of *A* on *Y*.
*M*	Mediator of *A* on *Y*.
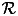	Denotes random treatment assignment.
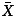	Sequential variables, e.g., 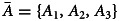 ; 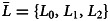 .
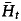	History of variables up to time *t* required for sequential exchangeability; more fully: 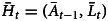 .
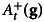	Used in dynamic treatment strategies to define a treatment under a dynamic treatment regime. 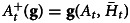 , where 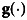 denotes a function such as: 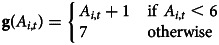 We abbreviate 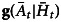 to 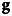 . This simplifies dynamic counterfactual expressions such as 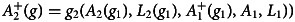
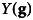	Potential outcome under the dynamic treatment strategy 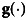 . Also represented as 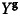 or 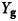 .
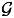	Denotes a causal graph, such as a causal directed acyclic graph (causal DAG), a Single World Intervention Template (SWIT), or a Single World Intervention Graph (SWIG).

**Table 2.** Elements of Causal Graphs

Symbol	Meaning	Example
**Graphical Notation**		
*X*	**Node or Vertex:** Variable denoted by a letter.	*A* (treatment), *Y* (outcome)
*X*_*t*_	**Time-indexed node:** Relative chronology.	*A*_1_ *Y*_2_
*X*_*ϕt*_	**Timing assumed** Relative chronology **asserted**.	*A*_*ϕ*1_ *Y*_*ϕ*2_
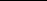	**Edge with no arrow:** Association.	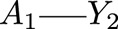
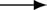	**Edge with an arrow:** Causal association.	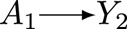
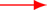	**Red arrow:** Path through which bias flows.	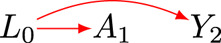
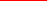	**Red association path:** Confounding path: ignores arrows to clarify statistical dependencies.	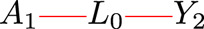
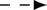	**Dashed arrow:** Causal effect not through a mediator (direct effect).	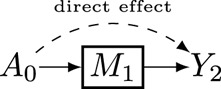
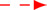	**Dashed red arrow:** Biased true effect, for example, when conditioning on a mediator.	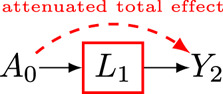
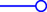	**Effect modification path:** Assumes 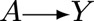 and focuses on the modification within levels of another variable. **Blue path is not evaluated**.	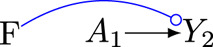
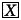	**Boxed variable:** Conditioning/adjustment.	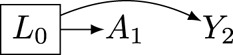
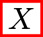	**Red boxed variable:** Variable that when conditioned upon induces bias.	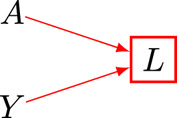
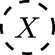	**Dashed circle:** No adjustment for variable.	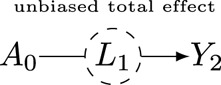
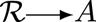	**Random treatment assignment:** Such that 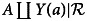 .	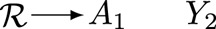
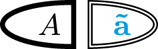	**Node Split**: Single World Intervention Graphs allow factorisation of counterfactuals by splitting a node at intervention with post-intervention nodes relabeled to match the treatment.	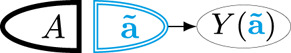
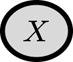	**Unobserved Node (SWIGs)**: *X*unmeasured.	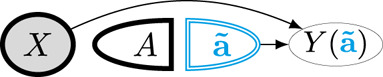
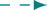	**Sequential dynamic treatments (modified treatment policy)**.	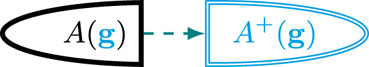
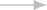	**Inactive path due to conditioning (SWIGs**)	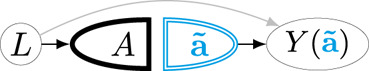

Throughout, for clarity, we repeat our definitions and graphical conventions as they are used. To begin, we define the following:

- *Node* – or equivalently a ‘variable’, denotes properties or characteristics of units within a population. In causal directed acyclic graphs, we draw nodes with respect to features in a *target population*, which is the population for whom we seek causal inferences (Suzuki et al., 2020). A time-indexed node, *X*_*t*_, allows us to index measurements within time intervals *t* ∈ 1…*T*, denoting relative chronology. If relative timing is not known, we may use *X*_*ϕt*_. The directions of arrows on a causal directed acyclic graph imply causation, and causation implies temporal order.
- Arrow (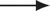
*)* – denotes a causal relationship from the node at the base of the arrow (a ‘parent’) to the node at the tip of the arrow (a ‘child’). In causal directed acyclic graphs, we refrain from drawing an arrow from treatment to outcome to avoid asserting a causal path from *A* to *Y*. Our purpose is to ascertain whether causality can be identified for this path. All other nodes and paths, including the absence of nodes and paths, are typically assumed.
- *Boxed variable* 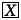 – denotes conditioning or adjustment for *X*.

Judea Pearl demonstrated that causal dependencies in a directed acyclic graph could be evaluated using observable probability distributions according to rules known as ‘d-separation’ (Pearl, 1995, 2009).

The rules, presented in Table 3, are as follows:

1. *Fork rule* 
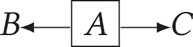
 –*B* and *C* are independent when conditioning on *A*: (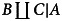).
2. *Chain rule* 
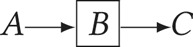
 – conditioning on *B* blocks the path between *A* and *C*: (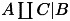).
3. *Collider rule* 
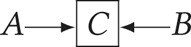
 –*A* and *B* are independent until conditioning on *C*, which introduces dependence (*A*
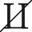
*B*|*C*.

From d-separation, Pearl derived a ‘backdoor adjustment theorem’, which provides a general identification algorithm given structural assumptions encoded in a causal directed acyclic graph Pearl (2009): in a causal directed acyclic graph (causal DAG), a set of variables *L* satisfies the backdoor adjustment theorem relative to the treatment *A* and the outcome *Y* if *L* blocks every path between *A* and *Y* that contains an arrow pointing into *A* (a backdoor path). Formally, *L* must:

1. not be a descendant of *A*; and
2. block all backdoor paths from *A* to *Y*.

If *L* satisfies these conditions, the causal effect of *A* on *Y* is identified by conditioning on 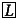 (Pearl, 2009). In what follows, I will assume that readers are familiar with causal directed acyclic graphs. Accessible introductions to causal directed acyclic graphs can be found in Pearl (2009), Barrett (2021), McElreath (2020), Neal (2020), Hernán and Robins (2024) and Bulbulia (2024a). (For an introduction to single world intervention graphs, used below, refer to Richardson & Robins, 2013a, b.)

**Table 3.** Five elementary causal structures in a causal directed acyclic graph

Five Elementary Causal Structures			
Structure	Causal DAG	Explanation	Implication
Two variables			
**1. Causality Absent**	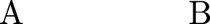	*A* and *B* have no causal effect on each other.	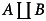
**2. Causality Present**	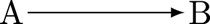	*A* causally affects *B*, and they are associated.	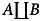
Three variables			
**3. Fork**	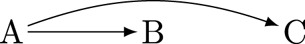	*A* causally affects both *B* and *C*; *B* and *C* are conditionally independent given *A*.	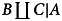
**4. Chain**	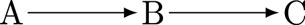	*C* is affected by *B* which is, in turn, affected by *A*; *A* and *C* are conditionally independent given *B*.	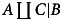
**5. Collider**	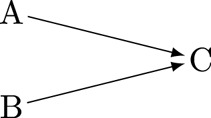	*C* is affected by both *A* and *B*, which are independent; conditioning on *C* induces association between *A* and *B*.	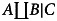

Key: 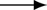, a directed edge, denotes causal association. The absence of an arrow denotes no causal association. **Rules of d-separation:** In a causal diagram, a path is ‘blocked' or ‘d-separated’ if a node along it interrupts causation. Two variables are d-separated if all paths connecting them are blocked or if there are no paths linking them, making them conditionally independent. Conversely, unblocked paths result in ‘d-connected’ variables, implying statistical association. Refer to Pearl (1995). Note that ‘d' stands for ‘directional’.

Implication: 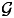 denotes a causal directed acyclic graph (causal DAG). *P* denotes a probability distribution function. Pearl proved that independence in a causal DAG (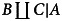)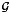 implies probabilistic independence (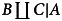)_*P*_; likewise if (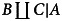)_*P*_ holds in all distributions compatible with 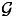, it follows that (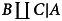)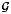 (refer to Pearl 2009, p. 61.) We read causal graphs to understand the implications of causality for relationships in observable data. However, reading causal structures from data is more challenging because the relationships in observable data are typically compatible with more than one (and typically many) causal graphs.

## Part 1: interaction as ‘effect modification’

We have said that in causal inference, we must explicitly define our causal question before applying statistical models to data. What question might the analysis of interaction answer? In causal inference, we think of interaction in two ways:

1. *Interaction as effect modification from a single intervention –* we want to understand how an intervention varies in its effect across the strata of the target population in which we are interested. For example, we might ask: does the one-year effect of attending weekly religious service differ among people born in Australia compared with people born in Egypt? Note that here we do not imagine intervening on birthplace.
2. *Interaction as joint intervention –* we want to understand whether the combined effects of two treatments administered together differ from the separate effect of each treatment acting alone. For example, we might ask: does the one-year effect of attending weekly religious service and the one-year effect of being at least one standard deviation above population average wealth differ from not attending any religious service and being at the population average in wealth? Here there are two interventions that might act individually, separately, or in concert.

Part 1 considers interaction as effect modification. Readers who do not wish to use the ‘effect modification’ may prefer the term ‘moderation’.

### Effect modification

First, we define the ‘sharp-null hypothesis’ as the hypothesis that there is no effect of the exposure on the outcome for any unit in the target population. Unless the sharp-null hypothesis is false, there may be effect modification (Bulbulia, 2024b). Clearly, the variability of causal effect modification cannot be assessed from descriptive measures of individuals in one's sample. Such heterogeneity must be modelled (refer to Tibshirani et al., 2024; Vansteelandt & Dukes, 2022). Alternatively, we might seek to compare whether the effects of treatments vary by strata within the target population. For example we may ask whether effects vary by culture group membership, gender or another grouping variable.

Table 4 describes conventions to clarify how to ask a causal question of effect modification. We assume no confounding of the treatment on the outcome and that *A* has been randomised (i.e. 
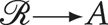
). As such, we will not use causal directed acyclic graphs to evaluate a treatment effect. We will assume 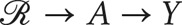.

**Table 4.** Graphical conventions we use for representing effect modification

Conventions for Effect Modification: We assume *A* 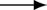 *Y*		
Symbol	Meaning	Example
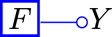	**Boxed blue variable and blue path:** observed effect-modification. **Blue arrow need not have a causal interpretation.**	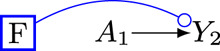
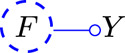	**Dashed blue circle and blue path:** effect-modifier not conditioned upon. **Blue arrow need not have a causal interpretation.**	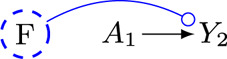

To sharpen focus on our interest in effect modification, we will not draw a causal arrow from the direct effect modifier *F* to the outcome *Y*. This convention is specific to this article. (Refer to Hernán & Robins, 2024: 126–127, for a discussion of ‘non-causal’ arrows.)

In Table 5 *G*_1_, we represent that *F* is a direct effect modifier for the effect of *A* on *Y*. The open arrow indicates that we are not attributing causality to *F*. Because our estimand does not involve intervening on *Z*, there is no need to close its backdoor paths. Note that if *F* were to affect *A*, we could still estimate the effect modification of *A* on *Y* because *F* has no causal interpretation. However, if *A* were to cause *F*, and *F* were to cause *Y*, then by the chain rule (recall Table 2 *G*_4_), conditioning on *F* would bias the effect estimate of *A* on *Y*.

**Table 5.** Effect Modification

Classification of Effect-Modification: Direct and Indirect		
No.	Type	Description
1	Measured variable F modifies effect: variation in the effect of 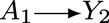 across levels of F, observed within the sample.	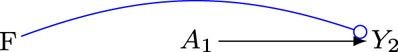
2	Unmeasured F modifies effect: potential variability in the effect of 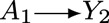 for unmeasured F; may be relevant to external validity.	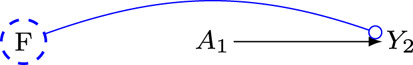
3	F is a direct effect-modifier: F directly alters the strength or direction of the effect of 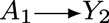 .	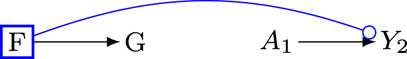
4	G is an indirect effect-modifier by proxy: when not conditioning on F, *G* is an indirect effect modifier of 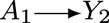 .	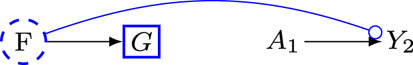
5	Conditioning on B makes G a surrogate effect-modifier: when not conditioning on F, conditioning on collider *B* opens a path from *G* to *Y* through F, making *G* and *B* indirect effect-modifiers of 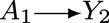 .	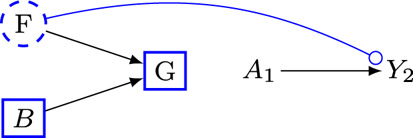
6	Only Z is a direct effect-modifier: conditioning on F renders its descendants independent of *Y* , leaving only F as the effect-modifier of 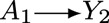 .	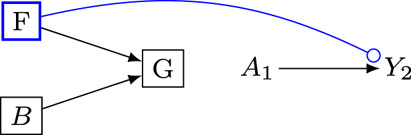

*A* denotes the treatment;

*Y* denotes the outcome;

*U* denotes an unmeasured confounder;

F denotes a direct-effect modifier;

{*G*, *B*} denote indirect effect modifiers of 
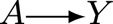
;

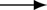
 asserts causality;

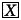
 indicates conditioning on variable *X*;

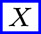
 indicates variables conditioned upon is an effect modifier (direct or indirect) of 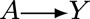;

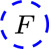
 indicates effect-modifier F is not conditioned upon;

Observation 1: classifying an indirect-effect modifier depends on the structure of causation and model specification.

Observation 2: Whether we condition on F or not, differences in the distribution of effect-modifiers within the sample population compared to the target population, specifically where these effect-modifiers interact with the causal path from 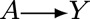, may introduce target validity bias. Because target-validity bias is indifferent to conditioning on F, we may represent effect modification without reference to whether F is included in the model: 
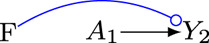

In Table 5 *G*_2_, we represent that *F* is an unobserved direct effect modifier of *A* to *Y*. When the distribution of direct effect modifiers *F* differs between two populations and effect modification is non-linear, marginal treatment effects between populations will generally differ and will not easily transport from one population to another. The concept of an average treatment effect has no meaning without a population over which the effect marginalises. This point, although obvious, has profound implications when investigators seek to assess whether their research generalises; refer to Hernán and Robins (2024) and Bulbulia (2024b). For example, if the study population differs in the distribution of features that modify a treatment effect, and no correction is applied, effect estimates will be biased for the target population in at least one measure of effect (Bulbulia, 2024b; Greenland, 2009; Lash et al., 2020)

We present two candidate effect modifiers in Table 5 *G*_3_. Notice that whether a variable is an effect modifier also depends on which other variables are included in the model. Here, *F* is a direct effect modifier and *G*, a descendant of *F*, is an indirect effect modifier. Suppose we were interested in whether treatment effects vary (on the difference scale) within levels of *F*. For example, let *F* denote childhood deprivation, *G* denote educational achievement, *A* denote a government educational initiative and *Y* denote recycling. If we were to condition on *F*, we would not observe effect modification by education *G* for the effect of the government initiative *A* on recycling behaviour *Y* : 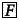 blocks the path 
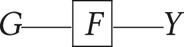
.

We present the same causal structure in Table 5 *G*_4_. However, we do not condition on the direct effect modifier *F*, but rather condition only on *G*, the indirect effect modifier. In this scenario, we would find that the effectiveness of the government initiative *A* on recycling behaviour *Y* varies by educational achievement *G*. Thus, we would observe *G* as an effect modifier because this path is open: 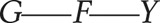.

In Table 5 *G*_5_, suppose we add another variable to our model, depression, denoted by *B*. We imagine *B* to be a stable trait or that investigators measured childhood depression (that is, *B* precedes *G*). Suppose we do not condition on the direct effect modifier *F* (childhood deprivation), but we condition on educational attainment (*G*) and depression (*B*). In this graph, *G* is a collider of *F* and *B*. Thus, conditioning on *G* (but not *F*) opens a path from 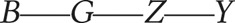. The investigators would find evidence for effect modification by depression on the effectiveness of the government intervention *A* on recycling (*Y*). However, they should not interpret this result to mean that if levels of depression were to change within the population the treatment effect would change. *B* is not causally related to *Y* in this scenario. Here, association is not causation.

In Table 5 *G*_6_, we will not find evidence for effect modification for *B* and *G* because conditioning on *F* blocks the flow of information that was open in *G*_4_ and *G*_5_. This again underscores the relativity of effect modification to (1) the structure of causality in the world and (2) an investigator's statistical modelling strategy.

These examples reveal the power – and simplicity – of causal diagrams to ‘transform the obvious’. Using causal directed acyclic graphs and Pearl's rules of d-separation, it is clear that the analysis of effect modification cannot be conducted without reference to an assumed causal order and an explicit statement about which variables within that order investigators have included in their statistical models (T. J. VanderWeele, 2012). Investigators and policymakers may make incorrect inferences and policy decisions if they do not understand the relevance of effect modification to such parameters. It is important to remember that when evaluating evidence for effect modification, we are not assessing the effects of intervening on variables other than the treatment. Instead, we qualitatively evaluate whether treatment effects vary across subgroups. For more on effect modification, refer to T. J. VanderWeele (2012), T. J. VanderWeele and Robins (2007) and Suzuki et al. (2013).

### Example showing scale dependence of effect modification

Suppose investigators are interested in whether treatment varies across levels of another variable, an effect modifier. We next illustrate how causal inferences about the presence or absence of effect modification depend on the scale that is used to measure the contrast. We show that an effect modifier on the ratio scale may not be an effect modifier on the difference scale, and vice versa.

Recall individual treatment effects are not observed. Assume a binary treatment is randomised, and we have *A* = *a* ∈ {0, 1}. Investigators are interested in comparing the magnitude of this treatment effect across two levels of *F* = *f* ∈ {0, 1}.

We define the average treatment effects for each group under each intervention as follows:
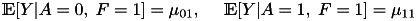

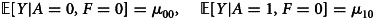


The treatment effect for each group on the difference scale (absolute scale) is given by:
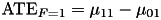

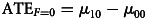


The treatment effect on the ratio scale (relative scale) for each group is:
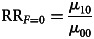

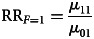


We say that there is effect modification on the difference scale if:
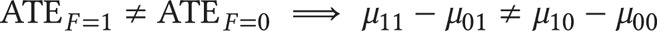


We say that there is effect modification on the ratio scale if:
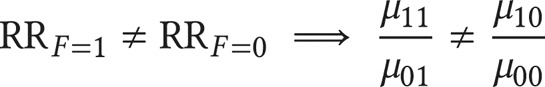


We have stated each causal question in relation to well-defined causal contrast and population, here defined by membership in *F*.

Imagine we obtain the following estimates from our study:

Outcomes under A = 0:

- *μ*_00_ = 5
- *μ*_01_ = 15

Outcomes under A = 1:

- *μ*_10_ = 10
- *μ*_11_ = 20

Next, we calculate the treatment effects on the difference and ratio scales for each group:

***Difference scale***


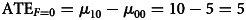




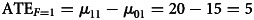




Both groups have the same treatment effect on the difference scale, ATE_*F*=0_ = ATE_*F*=1_ = 5. investigators conclude there is no evidence for effect modification on the difference scale.

***Ratio scale:***


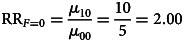




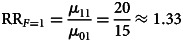




The treatment effect on the ratio scale is different between the two groups, RR_*F*=0_ = 2 ≠ RR_*F*=1_ ≈ 1.33. Hence, investigators find evidence for effect modification on the ratio scale.

The discrepancy in evidence for effect modification depending on the scale we choose arises because the two scales measure different aspects of the treatment effect: the absolute difference in outcomes vs. the relative change in outcomes. Parallel considerations apply to the analysis of interaction, where we imagine a joint intervention. For this reason, it is important to state the causal effect scale of interest in advance of estimation (Bulbulia, 2024a). We next consider interaction as a joint intervention.

## Part 2: interaction

### Introducing single world intervention graphs

When evaluating evidence for interaction, we must assess whether the combined effects of two treatments differ from the unique effects of each treatment relative to a baseline where neither treatment is administered. Understanding multiple interventions can be facilitated by using Single World Intervention Graphs (SWIGs) (Richardson & Robins, 2013a).

Single world intervention graphs employ Pearl's rules of d-separation but offer additional benefits by graphically representing the complex factorisations required for identification, presenting distinct interventions in separate graphs. The first advantage is *greater precision and clarity*: SWIGs allow us to consider identification conditions for each counterfactual outcome individually. Such precision is useful because identification conditions may differ for one, but not another, of the treatments to be compared. Node-splitting also makes it easier to determine identification conditions that are obscured in causal directed acylic graphs, for an example refer to supplementary materials S2. The second advantage is that *single world intervention graphs unify the potential outcomes framework with Pearl's structural causal model framework:* any causal relationship that can be represented in a causal directed acyclic graph can also be represented in a SWIG (Richardson & Robins, 2013b).

### Single world intervention graphs work by node-splitting

We create a single world intervention graph by ‘node-splitting’ at each intervention such that the random variable that is intervened upon is presented on one side and the level at which the random variable is fixed is presented on the other.

Consider a template graph Table 6 *G*. Applying node-splitting to *A* involves creating separate graphs for each value of *A* to be contrasted.

1. *SWIG for A* *= 0* – denoted as 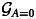, this graph shows the hypothetical scenario where *A* is set to 0.
2. *SWIG for A* *= 1* – denoted as 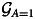, this graph shows the hypothetical scenario where *A* is set to 1.

**Table 6.** Single World Interventions Graphs 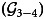 present separate causal diagrams for each treatment to be contrasted. A Single World Intervention Template 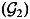 is a ‘graph value function’ that produces the individual counterfactual graphs (Richardson & Robins, 2013a). On the other hand, causal directed acyclic graphs, such as 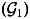, require positing interventional distributions. The formalism underpinning these interventional distributions is mathematically equivalent to formalism underpinning the potential outcomes framework, assuming the errors of the underlying structural causal models that define the nodes on which interventions occur are independent (Richardson & Robins, 2013a). Single World Intervention Graphs (SWIGs), however, permit the comparison of distinct interventions in our causal diagram without requiring that the non-parametric structural equations that correspond to nodes on a causal graph have independent error structures. This is useful when attempting to identify the causal effects of sequential treatments, refer to supplementary materials **S2**

Single World Intervention Graphs	
**Targeted Causal Contrast**	
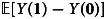	
**1. Causal Directed Acyclic Graph: assume interventions possible on each node**	
	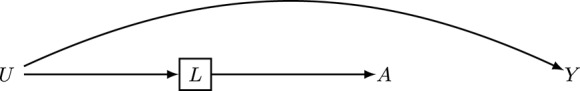
**2. Single World Intervention Template (SWIT): split-nodes at treatment, futures relabelled as counterfactual**	
	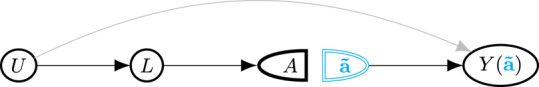
**3. Single World Intervention Graphs (SWIGs): factorisation of counterfactual outcomes using separate graphs, one for each treatment to be compared**	
	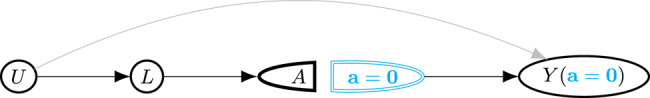
**4.**	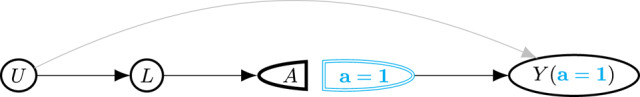

**Key**: 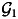 is a causal DAG: *Y* denotes the outcome; *A* denotes treatment; *U* denotes an unmeasured confounder. 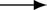 denotes an assumed causal path. 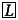 denotes conditioning on measured variables. Conditioning on *L* is sufficient to block the backdoor path between *A* and *Y*. We do not draw a path 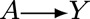 to underscore d-separation. Counterfactuals are not drawn on a causal DAG. We assume all nodes may be intervened upon. 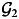 This represents a Single World Intervention Template (SWIT). The rules of d-separation remain; however, we factorise counterfactuals separately for each treatment by ’node-splitting’ at an intervention. This distinguishes the random intervention variable from the fixed intervention, and descendants under fixed interventions are relabelled using potential outcomes notation. For example, 
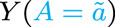
 is represented directly on our causal diagram.

To factorise potential outcomes, we must create separate Single World Intervention Graphs (SWIGs). Templates are ‘graph-valued functions’ that allow us to do this. Here, template 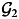 produces SWIG 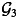 and SWIG 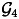. We use the convention 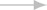 to denote closed confounding paths after conditioning on measured covariates and fixing interventions.

Assuming the structure of the world is faithfully captured in both causal DAG 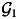 and the SWIGs 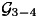, we find 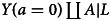 and 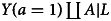

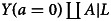

In these graphs, the node corresponding to the treatment *A* is split, relabelled with the random and fixed component, and then each node that follows is labelled with the fixed component until the next intervention. Here, 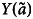
is the only variable to follow *A* and it is relabelled either *Y*(0) or *Y*(1) corresponding to whether *A* = 1 or*A* = 0; hence 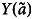 is relabelled as either *Y*(0) or *Y*(1). Note that we do not place both *Y*(0) and *Y*(1) on the same SWIG because the variables are not jointly observed. Hence, we evaluate identification for 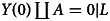 and 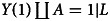 separately.

### Interaction as a joint intervention

We now use Single World Intervention Graphs (SWIGs) to clarify the concept of causal interaction as a joint intervention. Consider two treatments, denoted as *A* and *B*, and a single outcome, *Y*. Causal interaction as a joint interaction examines whether the combined effect of *A* and *B* on *Y* (denoted as *Y*(*a*, *b*)) differs from what would be expected from their individual effects. Specifically, we assess whether the effect of both treatments together is equal to, greater than, or less than the sum of their individual effects on the additive scale.

First, we obtain the expected outcomes when the entire target population is treated at each level of the treatments to be compared. These potential outcomes are illustrated in Table 7:

- Table 7 *G*_1_ – neither treatment *A* nor treatment *B* is given.
- Table 7 *G*_2_ – both treatment *A* and treatment *B* are given.
- Table 7 *G*_3_ – treatment *A* is given, and treatment *B* is not given.
- Table 7 *G*_4_ – treatment *A* is not given, and treatment *B* is given.

**Table 7.** 

Single World Intervention Graphs for Causal Interaction
**Targeted Causal Contrast For Interaction**
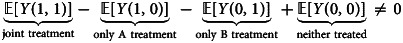
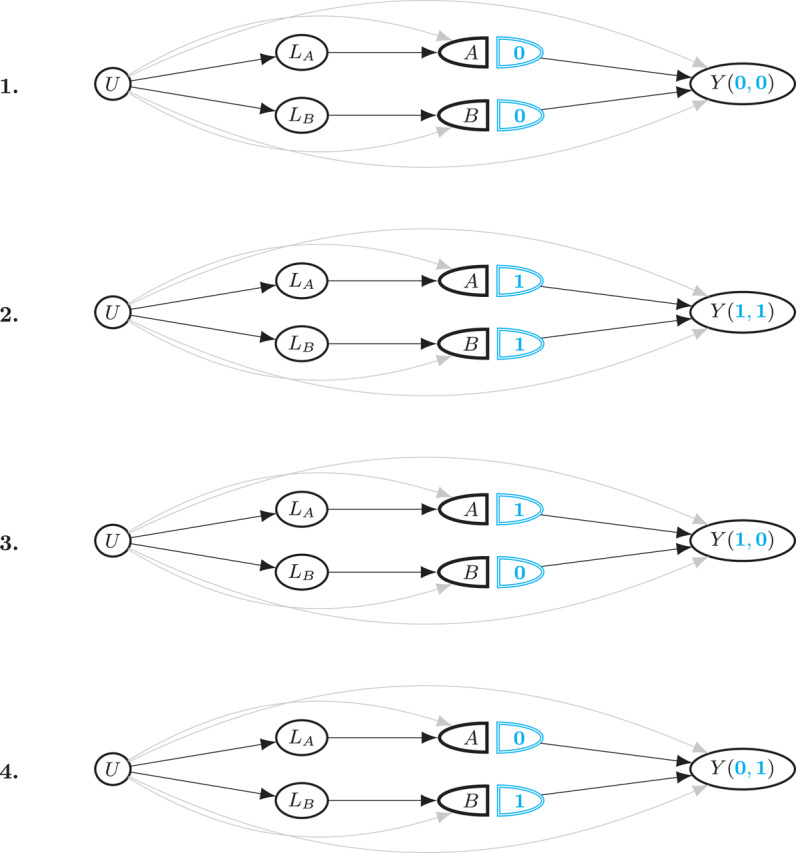

**Key**: *Y* denotes the outcome; *A* denotes treatment; 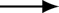 denotes an assumed causal path; 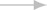 denotes blocked path by conditioning on *L*_*A*_ and *L*_*B*_

Single World Intervention Graphs show counterfactual states of the world under different interventions

To evaluate each intervention, we split a node into its random and deterministic components and represent descendants under fixed interventions using potential outcomes notation.

We require four counterfactual outcomes to evaluate interaction: joint treatment: 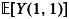; joint treatment: 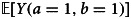; Only treat with A: 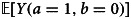; Only treat with B: 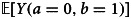; Treat neither with A nor with B: 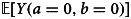.

By comparing these expected outcomes, we can determine the presence and nature of causal interaction between treatments *A* and *B* with respect to the outcome *Y*.

### Example

Consider the effect of beliefs in big Gods (exposure *A*) and a culture's monumental architecture (exposure *B*) on social complexity (outcome *Y*). Both interventions have equal status; we are not investigating effect modification of one by the other. The interventions must be well defined. We must state, understand and obtain measures for the quantities ‘big Gods’, ‘monumental architecture’ and ‘social complexity’ at specified time intervals after the interventions are first observed.

We need to state a population and scale to assess the individual and combined effects of *A* and *B*. Suppose our population consists of societies of primary urban genesis (Wheatley, 1971). Suppose further we seek evidence for causal interaction on the additive (difference) scale. Evidence for interaction would be present if the following inequality were to hold. Where,

- 𝔼[*Y*(1, 1)]– mean outcome for those jointly exposed to both treatments, big Gods and big architecture;
- 𝔼[*Y*(1, 0)] – mean outcome for those exposed only to the treatment of big Gods;
- 𝔼[*Y*(0, 1)] – mean outcome for those exposed only to the treatment of big architecture; and
- 𝔼[*Y*(0, 0)] – mean outcome for those exposed to neither treatment of big Gods nor of big architecture.

Evidence for interaction on the additive scale if
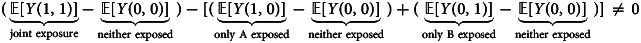


Simplifying:
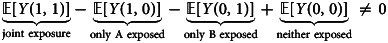


A positive value indicates evidence for a synergistic (super-additive) interaction. A negative value indicates evidence for a sub-additive interaction. A value close to zero implies no interaction on the additive scale.

Table 7 presents each counterfactual intervention. To identify these causal effects, we need to adjust for all confounders of the relationships between *A*, *B*, and *Y*. This includes any variables that influence both *A* and *Y*, both *B* and *Y*, both *A* and *B*, or all three variables simultaneously.

As with effect modification, evidence for causal interaction may differ depending on the measurement scale one chooses to assess it (T. J. VanderWeele, 2012; T. J. VanderWeele & Knol, 2014). For most policy settings, the additive scale is recommended because it directly relates to differences in outcome levels, which are often more actionable (see T. J. VanderWeele & Knol, 2014).

Note that if *A* and *B* potentially influence each other over time, we would need to collect longitudinal data and estimate causal effects using mediation analysis. Indeed, if there has been a co-evolution of religious culture, monumental architecture, and social complexity – as archaeologists have long reported (De Coulanges, 1903; Wheatley, 1971) – mediation analysis may be more appropriate. However, the requirements for causal mediation analysis are more stringent than those for causal interaction analysis, which we will consider next.

## Part 3: causal mediation analysis

In 1992, Robins and Greenland clarified the objectives of interpretable causal mediation analysis: to decompose the total effect into natural direct and indirect effects within a set of hypothetical interventions, contrasting their counterfactual outcomes (J. M. Robins & Greenland, 1992). This landmark paper has been to mediation analysis what *On The 0rigin of Species* has been to evolutionary biology (Darwin, 1859). However, mediation analysis in the human sciences remains rife with confusion. The primary source of this confusion is the application of statistical models to data without first defining the causal quantities of interest. **Associations derived from statistical mediation analysis do not necessarily imply causation and are typically uninterpretable**. This section considers how to formulate causal questions in mediation analysis.

### Defining a mediation analysis estimand

To understand causal mediation, we deconstruct the total effect into natural direct and indirect effects. Again, the total effect of treatment *A* on outcome *Y* is defined as the difference between potential outcomes when the treatment is applied vs. when it is not. The estimand for the total (or average, or ‘marginal’) treatment effect is given by:
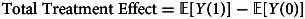

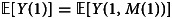


The total effect can be further decomposed into direct and indirect effects, addressing questions of mediation. The potential outcome *Y*(1), considering the mediator, expands to:

This considers the effect of the exposure *A* = 1 and the mediator at its natural value when *A* = 1. Similarly, the potential outcome 𝔼[*Y*(0)], considering the mediator, expands to:
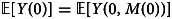


This quantity denotes the effect of exposure *A* = 0 and the mediator at its natural value when *A* = 0.

Next, we clarify our estimand by decomposing the total effect into the natural direct effect and the natural indirect effect.

The *natural direct effect* is the effect of the treatment on the outcome while maintaining the mediator at the level it would have been if the treatment had not been applied:
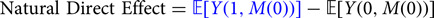


Here, the counterfactual quantities not directly realised in the data are highlighted in blue: 𝔼[*Y*(1, *M*(0))]. Notice we add this term to the potential outcomes when *A* = 0, recalling 𝔼[*Y*(0, *M*(0))] = *Y*(0).

The *natural indirect effect* is the effect of the exposure on the outcome that is mediated. To obtain these quantities, we compare the potential outcome *Y* under treatment, where the mediator assumes its natural level under treatment, with the potential outcome when the mediator assumes its natural value under no treatment:
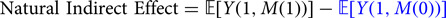


Here, the counterfactual quantities not directly realised in the data are again highlighted in blue: 𝔼[*Y*(1, *M*(0))]. Notice we subtract this term from the potential outcomes when *A* = 1, recalling 𝔼[*Y*(1, *M*(1))] = 𝔼[*Y*(1)].

By rearranging this decomposition, we find that the total effect is the sum of the natural direct effect and natural indirect effect. This is shown by adding and subtracting the term 𝔼[*Y*(1, *M*(0))] in our equation:
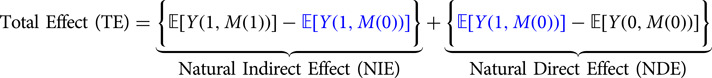


Table 8 presents a conceptual challenge for causal mediation analysis. Suppose we randomise a binary treatment *A* ∈ {0, 1}. Although randomising *A* does not ensure that there is no confounding of the mediator/outcome path, we assume no unmeasured confounding for either the treatment or the mediator. (We will relax this assumption in the next section.)

**Table 8.** In causal mediation, the quantities that we require to obtain natural direct and indirect effects, namely 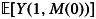, cannot be experimentally observed because we cannot treat someone and observe the level of their mediator if they were not treated

**Natural Direct and Indirect Effects in Causal Mediation**
**Targeted Causal Contrast For Natural Direct and Indirect Effects**
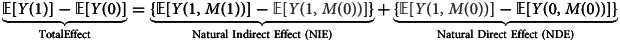
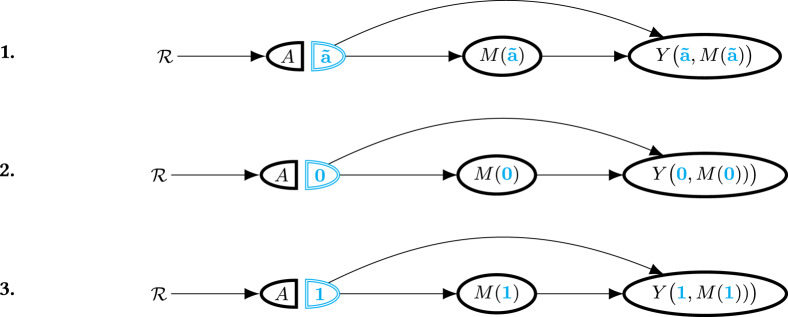

**Key**: *Y* denotes the outcome, *A* denotes treatment, and *M* denotes the mediator. 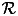 denotes randomisation into treatment, and 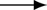 indicates an assumed causal path. For each intervention being contrasted, we split a node into its random and deterministic components and use potential outcomes notation to represent descendants under fixed interventions.

Table 8 *G*_1_ is a single world intervention Template (SWIT), which generates SWIGs for each condition.

Table 8 *G*_2_ presents counterfactual outcomes for condition *A* = 0; here, the natural value of *M* is *M*(*a* = 0), and the counterfactual outcome is given by *Y*(0, *M*(0)).

Table 8 *G*_3_ presents counterfactual outcomes for condition *A* = 1; here, the natural value of *M* is *M*(*a* = 1), and the counterfactual outcome is given by *Y*(1, *M*(1)).

These SWIGs clarify that we cannot identify natural direct and indirect effects from observations on individual units under treatment because 𝔼[*Y*(1, *M*(0))] is not observable (Shi et al., 2021; Steen et al., 2017; Valeri et al., 2014; T. J. VanderWeele, 2015; T. VanderWeele & Vansteelandt, 2014; Vansteelandt et al., 2012). Expressing these quantities requires a counterfactual framework. Here, we see that a counterfactual formulation of mediation analysis has made the familiar strange. However, under assumptions, we can sometimes recover natural direct and indirect effects from data (T. J. VanderWeele, 2015), given that our interest is in contrasts obtained for the target population, not for individuals, where we assume no causal effects are directly observed.

### Assumptions of causal mediation analysis

Table 9 *G*_1_ presents a Single World Intervention Template (SWIT) that specifies the assumptions required for inferring natural direct and indirect effects. This template highlights that, when estimating natural mediated effects, we only intervene on the treatment. Therefore, we must infer the mediated effect of the treatment under the condition that the mediator is set to the level it would naturally take under the control condition.

**Table 9.** Assumptions of Causal Mediation Analysis

Assumptions in Causal Mediation Analysis	
**For Natural Indirect and Direct Effects**	
**1.**	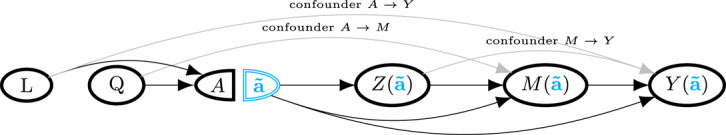
**2.**	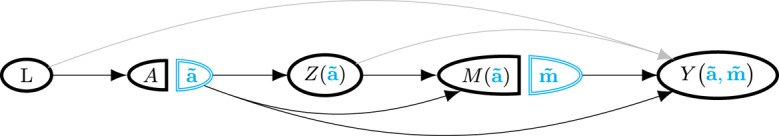

**Key**: *Y* denotes the outcome; *A* denotes treatment; *M* denotes the mediator; *L* is the measured confounder of the path *A* → *Y*; *Q* is the measured confounder of the path *A* → *M*; *N* is the measured confounder of the path *M* → *Y* ; 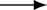 denotes a causal path; 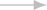 denotes a blocked backdoor path.

**The assumptions of causal mediation**

1. No unmeasured treatment-outcome confounder, hence we condition on *L*;

2. No unmeasured treatment-mediator confounder, hence we condition on *Q*;

3. No unmeasured mediator-outcome confounder, hence we condition on *Z*;

4. No mediator-outcome confounder affected by the exposure — a problem because we must not condition on *Z* to estimate the natural indirect effect.

**Randomisation only guaranteed assumptions 1 and 2** 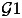: *Z* is a confounder of the *M* to *Y* path. We must, therefore, condition on *Z*. However *Z* is also a mediator of the *A* to *M* path. We must, therefore, not condition on *Z*. In this setting, there is no identification of natural indirect and direct effects of *A* through *M* because *Z* is both a confounder and an intermediary mediator. We may, however, estimate natural direct and indirect for the combined mediated effect of *M* and *Z*. We may use random draws from distributions of 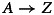 and *A to M* to obtain analogues of natural direct and indirect effects. 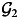: If assumptions 1 and 3 are satisfied, controlled direct effects can be identified in the presence of intermediary confounding. This requires fixing the mediator to some level *M* = *m* and evaluating the direct (unmediated) effect of treatment at this level of the mediator. However, in this setting, there is no longer a decomposition of the total effect into the natural direct and natural indirect effects for a controlled direct effect.

Additionally, Table 9 *G*_1_ also clarifies the assumptions needed for inferring controlled direct effects, where the mediator is fixed to a level specified by the investigators. In this scenario, we obtain causal contrasts by fixing variables to specific states.

Consider the hypothesis that cultural beliefs in ‘big Gods’ influence social complexity, with political authority mediating. Assuming that we have well-defined interventions and outcomes, what requirements are necessary to decompose this causal effect into natural direct and indirect effects?

1. *No unmeasured exposure-outcome confounder –* this requirement is expressed as 
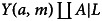
. After accounting for the covariates in set *L*, there must be no unmeasured confounders influencing cultural beliefs in big Gods (*A*) and social complexity (*Y*). For example, if our study examines the causal effect of cultural beliefs in big Gods on social complexity, and the covariates in *L* include factors such as geographic location and historical context, we need to ensure that these covariates effectively block any confounding paths between *A* and *Y*. The relevant path in Table 9 *G*_1_ is the confounder of the path *A* → *Y*.
2. *No unmeasured mediator-outcome confounder* – this requirement is expressed as 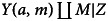. After controlling for the covariate set *Z*, we must ensure that no other unmeasured confounders affect political authority (*M*) and social complexity (*Y*). For instance, if trade networks affect political authority and social complexity, we must account for trade networks to block the path linking our mediator and outcome. The relevant path in Table 9 *G*_1_ is the confounder of the path *M* → *Y*.
3. *No unmeasured exposure-mediator confounder* – this requirement is expressed as: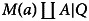. After controlling for the covariate set *Q*, we must ensure that no additional unmeasured confounders affect cultural beliefs in big Gods (*A*) and political authority (*M*). For example, the capability to construct large ritual theatres may influence the belief in big Gods and the level of political authority. If we have indicators for this technology measured before the emergence of big Gods (these indicators being *Q*), we must assume that accounting for *Q* closes the backdoor path between the exposure and the mediator. The relevant path in Table 9 *G*_1_ is the confounder of the path *A* → *M*.
4. *No mediator-outcome confounder affected by the exposure* – this assumption requires that there are no unmeasured confounders of the mediator-outcome relationship that are themselves affected by the exposure *A*. Such confounders cannot be adjusted for without introducing bias. The relevant path in Table 9 *G*_1_ involves confounders of the 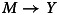 path that are influenced by *A*. Satisfying assumption 4 imposes considerable demands on causal mediation analysis. When the exposure influences a confounder of the mediator and outcome, we face a dilemma. Without adjusting for this confounder, a backdoor path between the mediator and the outcome remains open, introducing bias. In this setting, we cannot recover the natural direct and indirect effects from observational data. We may may need to settle for investigating controlled direct effects, estimate jointly mediated effects of *Z* and *M* together, or consider alternative estimands as suggested by VanderWeele and others (Díaz et al., 2023b; J. M. Robins & Richardson, 2010; T. J. VanderWeele et al., 2014; T. J. VanderWeele, 2015; T. J. VanderWeele & Tchetgen Tchetgen, 2017; Vo et al., 2024).

Notice that even when assumptions 1–4 are satisfied, natural direct effect estimates and natural indirect effect estimates require conceptualising a counterfactual that is never directly observed on any individual, namely: *Y*(1, *M*(0)). Such effects are only identified in distribution (refer to T. J. VanderWeele, 2015).

### Controlled direct effects

Consider another identification challenge, as described in template Table 9 *G*_1_. Suppose we aim to understand the effect of a stringent pandemic lockdown, *A*, on psychological distress, *Y*, focusing on trust in government, *M*, as a mediator. Further, suppose that pandemic lockdowns may plausibly influence attitudes towards the government through pathways that also affect psychological distress. For instance, people might trust the government more when it provides income relief payments, which may also reduce psychological distress.

Under the rules of d-separation, conditioning on income relief payments (*Z)* could block necessary causal paths. If we adjust for *Z*, we might block part of the effect of *A* on *M* and *Y* that operates through *Z*, potentially biasing the estimation of the natural indirect effect. The paths 
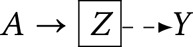
 and 
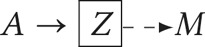
 are blocked. If we do not adjust for *Z*, *Z* acts as an unmeasured confounder of the 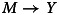 relationship since *Z* influences both *M* and *Y*. The path 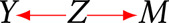. remains open, introducing bias.

In such a scenario, it would not be feasible to consistently decompose the total effect of the exposure (pandemic lockdowns) on the outcome (psychological distress) into natural indirect and direct effects. However, if all other assumptions were to hold, we might obtain an unbiased estimate for the controlled direct effect of pandemic lockdowns on psychological distress as a fixed level of government trust.

Table 9 *G*_2_ presents the weaker assumptions required to identify a controlled direct effect. We might examine the effect of the pandemic lockdown if we could intervene and set everyone's trust in government to, say, one standard deviation above the baseline, compared with fixing trust in government to the average level at baseline. We might use modified treatment policies (described below) that specify interventions as functions of the data. For instance, we might investigate interventions that ‘shift only those whose mistrust of government was below the mean level of trust at baseline and compare these potential outcomes with those observed’. Asking and answering precisely formulated causal questions such as this might lead to clearer policy advice, especially in situations where policymakers can influence public attitudes towards the government; see Williams and Díaz (2021), Díaz et al. (2023a) and Hoffman et al. (2022, 2023).

In any case, I hope this discussion of causal mediation analysis clarifies that it would be unwise to simply examine the coefficients obtained from statistical structural equation models and interpret them as meaningful. To answer any causal question, we must first state it, with respect to clearly defined counterfactual contrasts and a target population. Once we state our causal question, we find have no guarantees that the coefficients from statistical models are straightforwardly interpretable (T. J. VanderWeele, 2015).

For those interested in statistical estimators for causal mediation analysis, I recommend visiting the CMAverse website: https://bs1125.github.io/CMAverse/articles/overview.html (accessed 12 December 2023). This excellent resource provides comprehensive documentation, software and practical examples, including sensitivity analyses. Next, we will consider more complex scenarios that involve feedback between treatments and confounders across multiple time points – settings in which traditional statistical methods also fail to provide valid causal inferences.

## Part 4: time-fixed and time-varying sequential treatments (treatment strategies, modified treatment policies)

Our discussion of causal mediation analysis focused on how effects from two sequential treatments – the initial treatment and that of a mediator affected by the treatment – may combine to affect an outcome.

This concept can be expanded to investigate the causal effects of multiple sequential exposures, referred to as ‘treatment regimes’, ‘treatment strategies’ or ‘modified treatment policies’. Researchers often use longitudinal growth and multi-level models in many human sciences, where longitudinal data are collected. How may we identify such effects? What do they mean?

As before, to answer a causal question, we must first clearly state it. This involves specifying the counterfactual contrast of interest, including the treatments to be compared, the scale on which the contrast will be computed and the target population for whom inferences are valid. Without such clarity, our statistical models are often uninterpretable.

### Worked example: does marriage affect happiness?

Richard McElreath considers whether marriage affects happiness and provides a simulation to clarify how age structure complicates causal inferences (McElreath, 2020: 123–144). We expand on this example by first clearly stating a causal question and then considering how time-varying confounding invalidates the use of standard estimation methods such as multi-level regression.

Let *A*_*t*_ = 1 denote the state of being married at time *t* and *A*_*t*_ = 0 denote the state of not being married, where *t* ∈ {0, 1, *τ*} and *τ* is the end of the study. 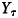 denotes happiness at the end of study. For simplicity, assume that this concept is well defined and measured without error.

Tables 10 and 11 reveal four treatment strategies and six causal contrasts that we may estimate for each treatment strategy combination.

**Table 10.** Four fixed treatment regimens in time-series data where exposure varies

Regime	Counterfactual outcome
Always married	*Y*(1, 1)
Never married	*Y*(0, 0)
Divorced	*Y*(1, 0)
Gets married	*Y*(0, 1)

**Table 11.** Six causal contrasts in time-series data where exposure varies

Comparison	Counterfactual outcome
Always married vs. Never married	𝔼[*Y*(1, 1) − *Y*(0, 0)]
Always married vs. Divorced	𝔼[*Y*(1, 1) − *Y*(1, 0)]
Always married vs. Gets married	𝔼[*Y*(1, 1) − *Y*(0, 1)]
Never married vs. Divorced	𝔼[*Y*(0, 0) − *Y*(1, 0)]
Never married vs. Gets married	𝔼[*Y*(0, 0) − *Y*(0, 1)]
Divorced vs. Gets married	𝔼[*Y*(1, 0) − *Y*(0, 1)]

To answer our causal question, we need to:

1. *Specify treatments –* define the treatment strategies being compared (e.g. always married vs. never married).
2. *Define the contrast* – state the counterfactual contrast of interest (e.g. 𝔼[*Y*(1, 1) − *Y*(0, 0)]).
3. *Identify the population* – specify the population for which the inferences are valid (e.g. adults aged 20–40).

### Time-varying confounding with treatment-confounder feedback

Table 12 *G*_1_ and Table 12 *G*_2_ represent two subsets of possible confounding structures for a treatment regime conducted over two intervals. Covariates in *L*_*t*_ denote measured confounders, and *U* denotes unmeasured confounders. *A*_*t*_ denotes the treatment, ‘Marriage Status,’ at time *t*. *Y* denotes ‘Happiness’ measured at the end of the study.

**Table 12.** Single World Intervention Graph for Sequential Treatments

Identification of Fixed and Dynamic Treatments Regimes
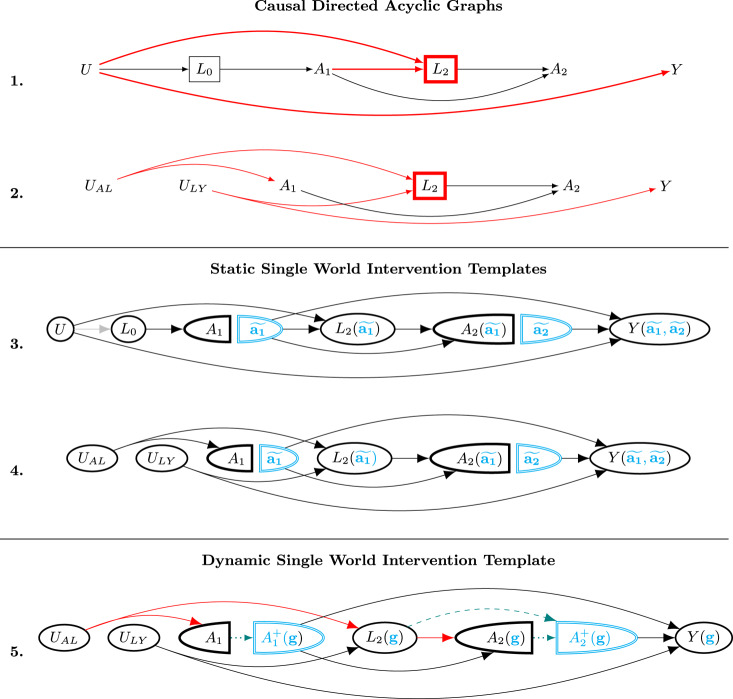

**Key**: *Y* represents the outcome, *A*_*t*_ the treatment, *U* unmeasured confounders, and *L*_*t*_ measured confounders ensuring 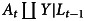. Researchers aim to determine if 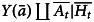, with *A*_*t*_ as a sequence of treatments and 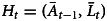 indicating past treatment and confounder histories. Counterfactual contrasts are predefined for static treatments and adaptive for dynamic treatments. 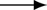 indicates causal paths; 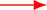 shows confounding paths; In Single World Intervention Graphs, 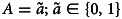; 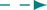 highlights dependencies from time-varying treatments (modified treatment policies) in dynamic Single World Intervention Graphs (or templates). 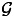
1–2 are causal directed acyclic graphs. All paths 
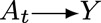
 are omitted. 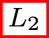 indicates conditioning on a confounder, which opens a backdoor path from treatment to outcome. 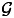
3–5, employ a Single World Intervention Template (SWIT), we apply d-separation to each intervention to be contrasted. We split each node into random and deterministic components, with descendants labelled as potential outcomes under treatment.

Consider the structure of confounding presented in Table 12 *G*_1_. To close the backdoor path from *A*_1_ to *Y*, we must condition on *L*_0_. To close the backdoor path from *A*_2_ to *Y*, we must condition on *L*_2_. However, *L*_2_is a collider of treatment *A*_1_ and unmeasured confounders, such that conditioning on *L*_2_ opens a backdoor path between *A*_1_ and *Y*. This path is highlighted in red: 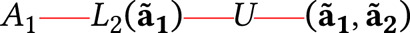.

If Table 12 *G*_1_ faithfully represents causality, it might seem that we cannot obtain valid inferences for any of the six causal contrasts we have defined. Indeed, using standard methods, we could not obtain valid causal inferences. However, J. Robins (1986) first described a consistent estimation function that can be constructed where there is time-varying confounding (Hernán et al., 2004; J. Robins & Hernán, 2008).

Table 12 *G*_3_ presents a single world intervention template that clarifies how identification may be obtained in fixed treatment regimes where there is time-varying confounding as observed in Table 12 *G*_1_. When constructing a single world intervention graph (or template), we obtain factorisations for counterfactual outcomes under a specific treatment regime by employing ‘node-splitting,’ such that all nodes following an intervention are relabelled as counterfactual states under the preceding intervention. After node-splitting, a fixed intervention is no longer a random variable. Thus, under fixed treatment regimes, the counterfactual states that follow an intervention are independent of the states that occur before node-splitting if there are no backdoor paths into the random partition of the node that has been split.

If all backdoor paths are closed into the random partitions of the nodes on which interventions occur, we can graphically verify that the treatment is independent of the counterfactual outcome for that intervention node. Where there are multiple interventions, we ensure sequential exchangeability at the following node – which we likewise split and relabel – by closing all backdoor paths between the random portion of the following treatment node. We have sequential independence if, for each intervention node, all backdoor paths are closed (refer to J. M. Robins & Richardson, 2010; Richardson & Robins, 2013b; Richardson & Robins, 2023).

The single world intervention template Table 12 *G*_3_ makes it clear that sequential identification may be obtained. *A*_1_ is d-separated from *Y* by conditioning on *L*_0_; *A*_2_ is d-separated from *Y* by conditioning on *L*_2_.

Notice that we cannot estimate the combined effect of a treatment strategy over *A*_1_ and *A*_2_ by employing regression, multi-level regression, statistical structural equation models or propensity score matching. However, special estimators may be constructed. (Refer to J. Robins, 1986, J. Robins & Hernán, 2008; Van Der Laan & Rose, 2011; Díaz et al., 2021; for recent reviews of special estimators refer to Hernán & Robins, 2024; Chatton et al., 2020; Van Der Laan & Rose, 2018; Chatton & Rohrer, 2024.)

### Time-varying confounding without treatment-confounder feedback

Consider how we may have time-varying confounding in the absence of treatment-confounder feedback. Suppose we are interested in computing a causal effect estimate for a two-treatment ‘marriage’ intervention on ‘happiness’. We assume that all variables are well defined, that ‘marriage’ can be intervened upon, that we have specified a target population, and that our questions are scientifically interesting. Here, we focus on the challenges in addressing certain causal questions with time-varying confounding without treatment confounder feedback. Table 12 *G*_1_ presents such a structure of time-varying confounding.

Let *U*_*AL*_ denote an over-confident personality, an unmeasured variable that is causally associated with decisions to marry early and with income. We do not suppose that *U*_*AL*_ affects happiness (*Y*). Taken in isolation, *U*_*AL*_ is not a confounder of the of the 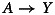 path.

Let *L*_*t*_ denote income. Suppose that income affects whether one stays married. For example, suppose it takes wealth to divorce. We can sharpen focus on risks of time-varying confounding by assuming that income itself does not affect happiness.

Let *U*_*AY*_ denote a common cause of income, *L*_2_, and of happiness at the end of the study, *Y*. An example of such a confounder might be educational opportunity, which affects both income and happiness. Table 12 *G*_2_ presents the confounding structure for this problem in its simplest form. To declutter, we remove the baseline node, *L*_0_, which we assume to be measured.

Notice that in this example there is no treatment–confounder feedback. We have not imagined that marriage affects wealth or even that wealth affects happiness. Nevertheless, there is confounding. To obtain valid causal inference for the effect of *A*_2_ on *Y*, we must adjust for *L*_2_ [otherwise: 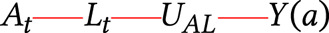]. However, *L*_2_is a collider of *U*_*AL*_ and *U*_*AY*_. In this setting, adjusting for *L*_2_ opens the path:
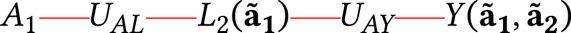


We have confounding without treatment-confounder feedback (refer to Hernán & Robins, 2024).

Table 12 *G*_4_ clarifies that sequential exchangeability can be obtained in the fixed treatment regime. To estimate the effect of *A*_2_ on *Y*, we must condition on *L*_2_. When estimating the effect of *A*_1_ on *Y*, all backdoor paths are closed because 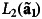 is a collider and . Note that because a single world intervention template does not represent the joint distributions of more than one treatment, treatment sequence or time-varying treatment, to evaluate the conditional independences we must specify the interventions of interest for *A*_1_ and *A*_2_. That is, we would need to evaluate at least two single world intervention graphs that correspond to the treatment level we wish to contrast. Note further that because there is time-varying confounding, we cannot use standard estimators such as multi-level regressions or structural equation models. Estimation must employ special stepwise methods (refer, for example, to Díaz et al., 2021; Williams and Díaz, 2021).

### Confounding under dynamic treatment strategies (modified treatment policies)

Suppose we are interested in the population average effect of divorce on happiness if divorce is only permitted for those with incomes above 1.5 times the average income. This is a dynamic treatment strategy because the treatment assignment depends on an individual's income, which might vary over time.

Define the treatment policy 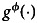 as remaining married for at least two additional years beyond baseline:
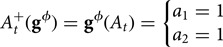


This regime is identified. The setting is identical to Table 12 *G*_2_ although no unmeasured variables and no arrow from *A*_1_ to *L*_2_.

However, every causal contrast requires a comparison of at least two interventions.

Define the treatment policy 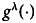 as divorce only if one's personal wealth is at least 50% greater than average and one would have divorced in the absence of intervention; otherwise, enforce marriage:
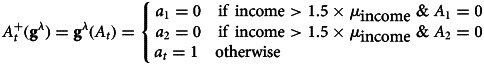


Notice that for the treatment policy 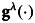, treatment is computed as a function of income at both the natural value of *A*_*t*_ and of wealth *L*_*t*_. Again, to declutter our graphs, we leave *L* at baseline off the graph, noting that adjustment at baseline does not change the confounding structure.

Template Table 12 *G*_5_ presents this confounding structure. To convey the dependence of the fixed node on covariate history under treatment, we use Richardson and Robins’ (2013a) conventions and draw a dashed line (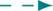) to indicate paths from the variables on which the time-varying treatment regime depends on the deterministic portion of the intervention node. This strategy clarifies that setting the treatment level requires information about prior variables, including the ’natural value of the treatment’ in the absence of any intervention (Young et al., 2014).

The reason for noting these dependencies on a Single World Intervention Graph is that such dependencies impose stronger identification assumptions. At every time *t*, *A*_*t*_ must be conditionally independent not only of the potential outcome at the end of the study but also of any future variable that might lead to a non-causal association between future treatments and the potential outcome at the end of the study. We clarify this additional requirement for *strong sequential exchangeability* in the next section.

### Identification of dynamic time-varying treatment strategies using an extension of Robin's dynamic G-formula

Supplementary materials S3 describes Richardson and Robins extension of J. Robins (1986) dynamic g-formula. Essentially, the algorithm can be stated as follows:

- *Step 1* – where 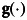 is a modified treatment policy, identify all the variables that influence the outcome 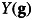, excluding those that are current or past treatment variables or covariates.
- *Step 2* – for each treatment at time *t*, check if the treatment is independent of the variables identified in Step 1, after accounting for past covariates and treatments, in each single world intervention graph where the treatment values are fixed. This step amounts to removing the dotted green arrows from the dynamic single world intervention graph in Table 12 *G*_5_, and doing so gives us Table 12 *G*_4_. For each time point, we recover a set of future counterfactual variables that includes the potential outcome for the treatment regime under consideration, 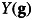, and other variables that the treatment might affect, including the natural value of future treatments. All backdoor paths must be closed to each member of this set of counterfactual variables. We call the more stringent assumptions required for identification in time-varying treatments (or equivalently longitudinal modified treatment policies; Díaz et al., 2023a) *strong sequential exchangeability*, where: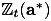 denotes the subset of vertices in 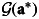 corresponding to 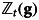;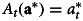 denotes the specific value of the treatment variable at time *t* under the intervention 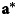;
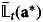

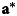
 denotes the set of covariates up to time *t* under the intervention 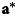; and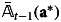 denotes the set of past treatment variables up to time *t* − 1 under the intervention 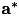.

Applying Richardson and Robins’ (2013a) dynamic extended g-formula, we obtain the following sets of future variables for which each current treatment must be independent:
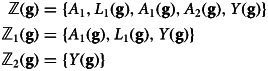


Having determined which variables must remain conditionally independent of each treatment in a sequence of dynamic treatments to be compared, we then consider whether strong sequential exchangeability holds. We do this by inspecting template Table 12 *G*_4_ (recall this is Table 12 *G*_5_ without the dashed green arrows). On inspection of *G*_4_ (the dynamic SWIG without dashed green arrows), we discover that this dynamic treatment strategy is not identified because we have the following open backdoor path:
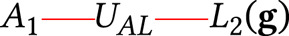


We also have:
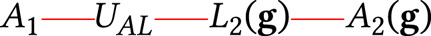


Strong sequential exchangeability fails for *A*_1_. We might consider lowering our sights and estimating a fixed or time-varying treatment strategy that can be identified.

Note that certain time-varying treatment strategies impose weaker assumptions than time-fixed strategies. For example, with a continuous intervention, we might consider intervening only if the observed treatment does not reach a specific threshold, such as:
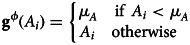


This is a weaker intervention than setting everyone whose natural value of treatment is above this threshold to precisely the threshold's value:
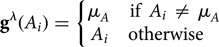


Whereas 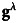 sets everyone in the population to the same treatment level, 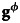
sets only those below a certain threshold to a fixed level but does not estimate treatment effects for those above (Hoffman et al., 2023). We can also write stochastic treatment functions (Díaz et al., 2021; Muñoz & Van Der Laan, 2012; T. VanderWeele & Vansteelandt, 2014; Young et al., 2014); see supplementary materials S4.

Of course, the details of every problem must be developed in relation to the scientific context and the practical questions that address gaps in present science. However, causal inference teaches us that the questions we ask – seemingly coherent and tractable questions such as whether marriage makes people happy – demand considerable scrutiny to become interpretable. When such questions are made interpretable, causal inference often reveals that answers may elude us, regardless of the quality and abundance of our data, or even if we randomise interventions. Modest treatment functions, however, might be more credible and useful for many scientific and practical questions. Such functions often cannot be estimated using the models routinely taught in the human sciences, such as multi-level modelling and statistical structural equation modelling.

## Conclusions

Philosophical interests in causality are ancient. Democritus once declared, ‘I would rather discover one cause than gain the kingdom of Persia’ (Freeman, 1948). Hume provided a general account of causality by referencing counterfactuals: ‘where, if the first object had not been, the second never would have existed’ (Hume, 1902). However, it was not until Jerzy Neyman's master's thesis that a quantitative analysis of causality was formalised (Splawa-Neyman, 1990). Remarkably, Neyman's work went largely unnoticed until the 1970s, when Harvard statistician Donald Rubin formalised what became known as the ‘Rubin Causal Model’ (also the Rubin–Neyman Causal Model) (Holland, 1986; Rubin, 1976).

In 1986, Harvard statistician James Robins extended the potential outcomes framework to time-varying treatments, laying the foundation for powerful new longitudinal data science methods (J. Robins, 1986). Judea Pearl introduced directed acyclic graphs, making identification problems transparent and accessible to non-specialists (Pearl, 1995). Robins and Richardson extended Pearl's graphical models to evaluate counterfactual causal contrasts on graphs, building on Robins’ earlier work. Concurrently, the causal revolution in economics opened new, fertile frontiers in causal data sciences. By the early 2000s, targeted learning frameworks were being developed (Van Der Laan & Rose, 2011), along with causal mediation analysis methods (Díaz et al., 2023b; Pearl, 2009; J. M. Robins & Greenland, 1992; Rudolph et al., 2024; T. J. VanderWeele, 2015; T. VanderWeele & Vansteelandt, 2014; Vansteelandt et al., 2012) and techniques for analysing time-varying treatments (Muñoz & Van Der Laan, 2012; Richardson & Robins, 2013a, 2023; J. Robins, 1986; J. M. Robins et al., 1999; J. Robins & Hernán, 2008; Shpitser et al., 2022; Young et al., 2014).

Readers should note that the causal inference literature contains vigorous debates at the horizons of discovery. However, there is a shared consensus about the foundations of causal inference and a common conceptual and mathematical vocabulary within which to express disagreements and accumulate progress – a hallmark of a productive science. Old debates resolve and new debates arise, the hallmark of a vibrant science.

Despite the progress and momentum of the causal revolution in certain human sciences, many areas have yet to participate and benefit. The demands for researchers to acquire new skills, coupled with the intensive requirement for data collection, have significant implications for research design, funding and the accepted pace of scientific publishing. To foster essential changes in causal inference education and practice, the human sciences need to shift from a predominantly output-focused, correlation-reporting culture to a slow, careful, creative culture that promotes retraining and funds time-series data collection. Such investments are worthwhile. Much as Darwin's theory transformed the biological sciences from speculative taxonomy, causal inference is slowly but steadily transforming the human sciences from butterfly collections of correlations to causal inferential sciences capable of addressing the causal questions that animate our curiosities.

## Supporting information

Bulbulia supplementary material Bulbulia supplementary material

## Supplementary material

To view supplementary material for this article, please visit https://doi.org/10.1017/ehs.2024.32

## Research transparency and reproducibility

No data were used in this paper.
