# Supplementary material for: Methods in causal inference. Part 2: Interaction, mediation, and time-varying treatments
Source: Evol Hum Sci. 2024 Oct 1;6:e41. doi: 10.1017/ehs.2024.32 (PMC11588565; doi:10.1017/ehs.2024.32)
Supplement: Bulbulia supplementary material [file S2513843X2400032Xsup001.zip › s1-part-2-time-vary.pdf]

Supplementary files for "Methods in Causal Inference Part 2: Interaction, Mediation, and Time-Varying Treatments"

Joseph A. Bulbulia[1]

[1] Victoria University of Wellington, New Zealand ORCID 0000-0002-5861-2056

2024-06-20

Table of contents

S1. Glossary . . . 2
S2. Single World Intervention Graphs Eludicate Complex Identification Problems . . . 4
S3. Richardson and Robin's Extended Dynamic G-formula . . . 6
S4. Structural Causal Models and Shift interventions . . . 8
References . . . 10

List of Tables

1 Glossary . . . 3
2 On the limitations of causal directed acyclic graphs compared to Single World Intervention Graphs. . . . 5

S1. Glossary

Table 1: Glossary

Term	Definition
**Acyclic**	No variable can be an ancestor or descendant of itself on a causal graph.
**Adjacent Nodes**	Two nodes connected by an arrow are adjacent.
**Adjustment Set**	Variables conditioned to block all backdoor paths between treatment (A) and outcome (Y).
**Ancestor/Descendants**	Nodes connected by directed edges. All descendants of an ancestor can be reached by directed paths.
**Arrow**	Represents direct causation in a causal diagram, pointing from cause to effect.
**Average Treatment Effect (ATE)**	The difference in expected outcomes between treated and untreated units across a specified population. Synonym for Marginal Effect.
**Backdoor Path**	Path that, if not blocked, may associate the treatment and outcome without causality.
**Causal Contrast**	The difference in expected outcomes under different treatment levels.
**Causal Contrast Scale**	The metric for quantifying causal contrasts, chosen based on outcome type and research question.
**Causal Diagram (Causal DAG)**	A graph representing causal relationships to evaluate an identification problem; must be acyclic and describe all confounding, measured and unmeasured for the target population.
**Causal Estimand**	The causal contrast of interest in a study; specifies the intervention, outcome, contrast scale, and target population; stated before analysis.
**Causal Path**	Asserts a change in the parent node will induce a change in its child.
**Censoring**	the sample population is not representative of the target population at baseline (left censoring) or is no longer representative at the end of study (right censoring).
**Collider/"Immorality"**	A variable where two causal paths meet head-to-head, may induce non-causal associations between its parents.
**Conditional Average Treatment Effect (CATE)**	The treatment effect for specific subgroups, defined by measured characteristics.
**Conditioning**	Adjustment for variables in analysis to distinguish causal effects from associations.
**Confounding**	Treatment and outcome are associated independently of causality or are disassociated despite causality, relative to the causal question.
**Confounder**	A variable or set of variables form part of an ideal identification strategy to reduce or eliminate confounding.
**Counterfactual or Potential outcomes**	Hypothetical outcomes under different treatment conditions to be contrasted, only one may be realised for each observed unit.
**Direct Effect (Natural Direct Effect)**	The difference between potential outcomes when the treatment is applied and the mediator is set to no-treatment versus when neither the treatment nor the mediator is applied.
***d*-separation**	Backdoor paths are blocked, satisfying the assumption of 'no unmeasured confounding'.
**Descendant (Child)**	A node causally influenced by a prior node (Parent). A child is a parent's direct descendant.
**Effect-Measure Modifier/Effect-Modifier**	A variable that affects the magnitude or direction of a causal effect.
**Estimator**	Algorithm to compute a statistical estimand from data.
**External Validity/Target Validity**	The generalisability of study findings to the prespecified target population; assumes internal validity.
**Factorisation**	Decomposing the joint probability distribution of variables into a product of conditional probabilities of each variable given its parents.
**Heterogeneous Treatment Effects**	Variation in treatment effects across subgroups or contexts.
**Identification Problem**	Ensure no unmeasured confounding.
**Incident Exposure Effect**	Causal effect of initiating a new treatment.
**Indirect Effect (Natural Indirect Effect)**	The average difference in potential outcomes when the mediator is at its natural value under treatment versus no treatment.
**Instrumental Variable**	Associated with treatment but affecting the outcome only through the treatment, used for estimating causal effects amidst confounding.
**Intention-to-Treat Effect**	The effect of treatment assignment, what random assignment obtains.
**Internal Validity**	The extent to which causal associations in the study population are accurately identified.
**Inverse Probability of Censoring Weights**	Weights used to adjust for bias due to attrition in longitudinal studies.
**Inverse Probability of Treatment Weights**	Weights that create a pseudo-population to achieve treatment balance across conditions.
**Local Markov Assumption**	assumption that a variable is independent of its non-descendants given its immediate parents in a causal graph.
**Longitudinal Study/Panel Study**	A research design that repeatedly tracks and measures the same units over time.
**Loss-to-follow-up**	Participant attrition.
**Markov Assumption**	assumption that a variable is independent of its non-descendants given its parents in a causal graph
**Marginal Effect**	Synonym for Average Treatment Effect.
**Measurement Error Bias**	Bias introduced when measurements of variables are inaccurately recorded, either through correlated or direct measurement errors, or when uncorrelated errors mask the true effects.
**Mediator**	A variable through which a treatment affects an outcome.
**Modularity Assumption**	Interventions on one set of variables do not directly alter the conditional distribution of other variables, given their direct causes.
**Node**	Represents a variable in a causal diagram, also called "Vertex"
**Observational Study**	Treatment assignment is not controlled by the investigator.
**Parent/Child**	Adjacent nodes connected by a directed path.
**Path**	Nodes are connected by a sequence of edges. Directed paths follow directed edges.
**Per-Protocol Effect**	The causal effect under full-treatment adherence.
**Prevalent Exposure Effect**	Effect of current or ongoing treatments.
**Propensity Score**	The probability of receiving a treatment based on observed characteristics used for confounding adjustment in observational studies.
**Randomised Treatment Assignment**	Chance treatment assignment.
**Randomised Controlled Trial (RCT)**	Uses random treatment assignment to balance confounders across the treatments to be compared.
**Reverse Causation**	Mistaking the effect for the cause in an analysis.
**Sample Weights**	Adjusts sample data to represent the target population in analysis better.
**Selection Bias**	Systematic errors from non-representative study participation or attrition affecting generalisability.
**Sequentially Treatment**	multiple treatments may be fixed our time-varying
**Single World Intervention Graph (SWIG)**	A graph to obtain causal identification under a single counterfactual treatment regime by splitting nodes into random and fixed components, where the fixed inherits edges directed into the node (parents) and the random inherits edges out (children).
**Single World Intervention Template (SWIT)**	A graph-valued function or template generates SWIGs (is not itself a graph).
**Statistical Estimand**	The parameter of interest in a statistical model, not necessarily causal.
**Statistical Estimate**	The value obtained for a statistical estimand from data analysis.
**Statistical Model**	Describes covariance between variables; without structural assumptions, statistical models do not identify causal effects.
**Structural Model**	Assumptions about causal relationships encoded in diagrams, essential for identifying causality from statistical associations.
**Study Population**	The population from which data are collected, also called the "sample population."
**Target Population**	The broader population to which study results are intended to apply.
**Target Trial**	An observational study emulating an ideal experiment by pre-specifying a causal estimand, eligibility criteria, and data ordering for an incident exposure effect.
**Time-Varying Confounding**	Confounding that changes over time, complicating causal effect estimation using standard methods.
**Total Effect**	The difference in mean potential outcomes under contrasted treatments in a study.

S2. Single World Intervention Graphs Eludicate Complex Identification Problems

According to Pearl (2009), example 11.3.3. $Y(x_0, x_1)$ is not independent of X_1 given Z and X_0. Template $\mathscr{G}_2$ and Single World Intervention Graphs $\mathscr{G}_2 - 6$ examine counterfactual independence. Counterfactual nodes are obtained by node-splitting. $\longrightarrow$ denotes a backdoor path that is closed when a treatment is fixed. $\longrightarrow$ highlights identifying paths for $X_0 = x_0$ and $X_1 = x_1$. J. M. Robins & Richardson (2010) uses a variation of $\mathscr{G}_2$ to show that there is sequential exchangeability of $X_t \forall t\ :\ Y(x_1, x_0) \coprod X_0$ (unconditionally) and $Y(x_1, x_0) \coprod X_1(x_0)|Z(x_0), X_0$. By causal consistency $Z(x_0) = Z|X_0 = x_0$. Single world interventions $\mathscr{G}_{3-6}$ make this sequential exchangeability clear (refer to Richardson & Robins (2013)). Such clarity is, in my view, an excellent reason to use Single World Intervention Graphs.

Table 2: On the limitations of causal directed acyclic graphs compared to Single World Intervention Graphs.

Single World Intervention Graphs Make Challenging Identification Problems Clear

Pearl 2009 claims $Y(x_0, x_1) \coprod X_1|Z, X_0 = x_0$ is not identified.

1

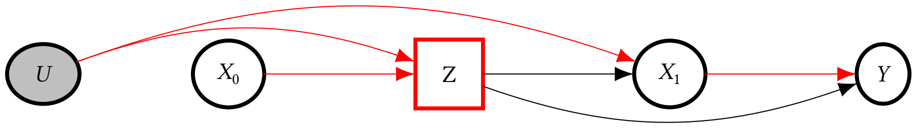

Single World Intervention Template reveals sequential exchangeability for all fixed strategies in $\bar{X}$

2

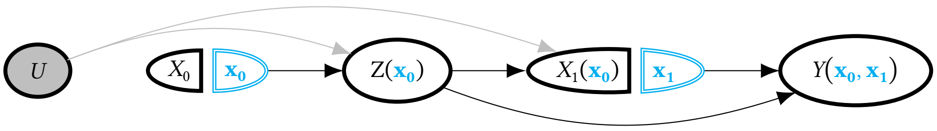

Single World Intervention Graphs: sequential exchangeability by each counterfactual

3

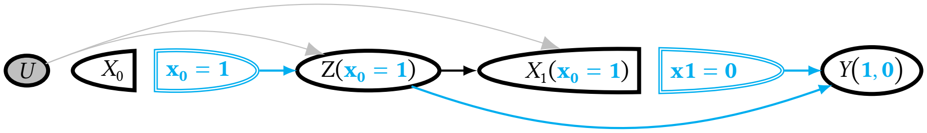

4

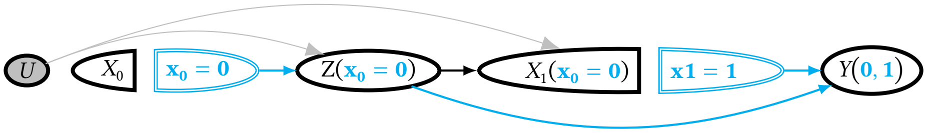

5

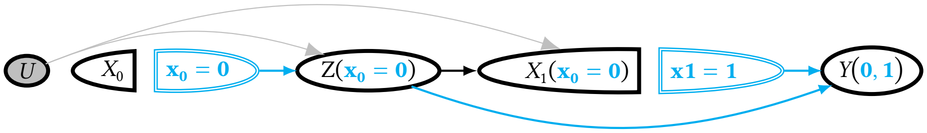

6

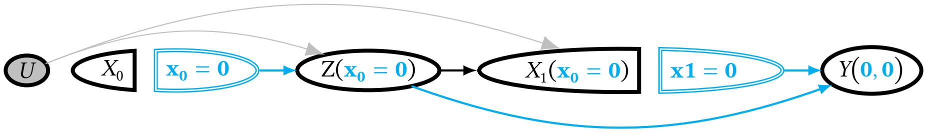

Key: $\mathcal{G}1$: Y denotes the outcome; U denotes an unmeasured confounder; X_t denotes intervention at time $t \in \{0, 1\}$. $\boxed{Z}$ denotes mediator of X_0 and X_1. - -► indicates the claimed bias path.

S3. Richardson and Robin's Extended Dynamic G-formula

Richardson & Robins (2013) propose an extension of J. Robins (1986)'s dynamic g-formula for identifying causality under dynamic treatment regimes. Here, I reproduce the key details of their identification algorithm. I refer readers to Richardson & Robins (2013) for the full algorithm and its proofs.

First, define the set of counterfactual variables in our dynamic Single World Intervention Graph (or Template):

$\mathbb{A}^+(\mathbf{g})$: denotes the set of modified treatment variables under a dynamic regime $\mathbb{V}(\mathbf{g})$: denotes the set of counterfactual nodes following treatments. $\mathbb{W}(\mathbf{g})$: denotes the combined set of all counterfactual variables under a dynamic regime corresponding to Table 12 $\mathbf{G}_4$ in the main article.

Richardson & Robins (2013) define this set as follows:

$$\mathbb{W}(\mathbf{g}) \equiv \mathbb{A}^+(\mathbf{g}) \cup \mathbb{V}(\mathbf{g})$$

Next, at each intervention node t, our task is to find all ancestors of $Y(\mathbf{g})$ in $\mathbb{W}(\mathbf{g})$ that are not in the set of current or past treatment covariates. Richardson & Robins (2013) define this set as follows:

$$\mathbb{Z}_t(\mathbf{g}) \equiv \text{an}_{\mathcal{G}(\mathbf{g})}(Y(\mathbf{g})) \setminus (\mathbb{L}_t(\mathbf{g}) \cup \mathbb{A}_t(\mathbf{g}) \cup \mathbb{A}^+(\mathbf{g}))$$

Third, our task is to map $\mathbb{Z}$ to a new Single World Intervention Graph $\mathcal{G}(\mathbf{a}^*)$, where the intervention $\mathbf{a}^*$ is a specific value of $A = a$ assigned under $f^g(\cdot)$.

This new `dSWIG` or `dynamic Single World Intervention Graph` $\mathcal{G}(\mathbf{a}^*)$ is simply the original dSWIG $\mathcal{G}(\mathbf{g})$ with the dashed arrows removed.

Fourth, our task is to ensure conditional independence of the treatment $A_t = a^*$ with members of the set $\mathbb{Z}_t$, for all $\mathbf{a}^*$ (fixed nodes) and all time points $t \in 1...\tau$, where τ is the end of the study. Richardson & Robins (2013) define this set as follows:

$$\mathbb{Z}_t(\mathbf{a}^*) \coprod I(A_t(\mathbf{a}^*) = a_t^*) \mid \bar{\mathbb{L}}_t(\mathbf{a}^*), \bar{\mathbb{A}}_{t-1}(\mathbf{a}^*) = \bar{\mathbf{a}^*}_{t-1}$$

where the authors use I to denote the indicator function:

$$I(A_k(\mathbf{a}^*) = a_t^*) = \begin{cases} 1 & \text{if } A_k(\mathbf{a}^*) = a_t^*, \\ 0 & \text{otherwise.} \end{cases}$$

where:

- $\mathbb{Z}_t(\mathbf{a}^*)$: denotes the subset of vertices in $\mathcal{G}(\mathbf{a}^*)$ corresponding to $\mathbb{Z}_t(\mathbf{g})$.
- $A_t(\mathbf{a}^*) = a_t^*$: denotes the specific value of the treatment variable at time t under the intervention $\mathbf{a}^*$.
- $\bar{\mathbb{L}}_t(\mathbf{a}^*)$: denotes the set of covariates up to time t under the intervention $\mathbf{a}^*$.
- $\bar{\mathbb{A}}_{t-1}(\mathbf{a}^*)$: denotes the set of past treatment variables up to time $t-1$ under the intervention $\mathbf{a}^*$.

In the example describe in the main article Table 12 $\mathcal{G}_4$, $\mathbb{Z}_t(\mathbf{g})$, we apply Richardson & Robins (2013)'s dynamic extended g-formula, and obtain:

$$\begin{aligned} \mathbb{Z}(\mathbf{g}) &= \{A_1, L_1(\mathbf{g}), A_1(\mathbf{g}), A_2(\mathbf{g}), Y(\mathbf{g})\} \\ \mathbb{Z}_1(\mathbf{g}) &= \{A_1(\mathbf{g}), L_1(\mathbf{g}), Y(\mathbf{g})\} \\ \mathbb{Z}_2(\mathbf{g}) &= \{Y(\mathbf{g})\} \end{aligned}$$

Then we check conditional independencies for each treatment in the main manuscript Table 12 $\mathscr{G}_4$ (which is Table 12 $\mathscr{G}_5$ without the dashed green arrows). We inspect this template and learn the dynamic treatment strategy under consideration is not identified.

S4. Structural Causal Models and Shift interventions

Below is Díaz et al. (2021)'s formulation of Pearl (2009)'s mathematical representation of a structural causal model with non-independent errors. I present Díaz et al. (2021)'s formulation because it allows us to present a structural causal model for dynamic treatment strategies Richardson & Robins (2013), also known as longitudinal modified treatment policies Hoffman et al. (2023). Note further that Richardson & Robins (2013) develop their account of time-varying treatments using structural causal models. The difference is that on the potential outcome framework, identification does not rely on non-independent error terms or so-called 'cross-world' assumptions (Richardson & Robins, 2013, pp. 60–84).

Following Díaz, we begin by defining the sequence of variables in our model:

$$S_i = (W, Y_0, L_1, A_1, L_2, A_2, ..., L_\tau, A_\tau, Y_\tau) \sim \mathbf{P}$$

where S_i is a sample from the distribution $\mathbf{P}$ and includes baseline covariates W, intermediate outcomes L_t, treatments A_t, and final outcomes Y_τ over time periods $t = 1, 2, \ldots, \tau$.

We define the final outcome:

$$Y = A_{\tau+1}$$

We define the history of all variables up to treatment A_t as:

$$H_t = (\bar{A}_{t-1}, \bar{L}_t)$$

Here, $\bar{A}_{t-1}$ represents the history of treatments up to time $t-1$, and $\bar{L}_t$ represents the history of intermediate outcomes up to time t.

We define the vector of exogenous variables (error terms). Note on Pearl's structural causal model account, but not the potential outcomes framework, the error terms must always be independent:

$$U = (U_{L,t}, U_{A,t}, U_Y : t \in \{1 \ldots \tau\})$$

Where U describes the set of exogenous variables affecting L_t, A_t, and Y.

We assume the following deterministic functions for the intermediate outcomes, treatments, and final outcome:

1. For intermediate outcomes:

$$L_t = f_{L_t}(A_{t-1}, H_{t-1}, U_{L,t})$$

2. For treatments:

$$A_t = f_{A_t}(H_t, U_{A,t})$$

3. For the final outcome:

$$Y = f_Y(A_\tau, H_\tau, U_Y)$$

Longitudinal modified treatment policies (LMTPs) are defined as functions that assign treatments flexibly based on individual co-variate histories. Note that where there are multiple treatments, these histories will be partially counterfactual.

We replace the deterministic function for treatments:

$$A_t = f_{A_t}(H_t, U_{A,t})$$

With the intervention function:

$$A(\mathbf{g}_t)$$

On the structural causal model account, this intervention produces counterfactual histories given:

$$L_t(\bar{A}(\mathbf{g})_{t-1}) = f_{L_t}(A(\mathbf{g})_{t-1}, H_{t-1}(\bar{A}(\mathbf{g})_{t-2}), U_{L,t})$$

For treatments, the counterfactual variable $A_t(\bar{A}^{\mathbf{g}}_{t-1})$ is defined as the natural value of the treatment, i.e., the value of the treatment that would have been observed at time t under the intervention history leading up to it at $t-1$, and then discontinued:

$$A_t(\bar{A}^{\mathbf{g}}_{t-1}) = f_{A_t}(H_t(\bar{A}^{\mathbf{g}}_{t-1}), H_{t-1}(\bar{A}^{\mathbf{g}}_{t-2}), U_{L,t})$$

When all variables are intervened on, the counterfactual final outcome is:

$$Y(\bar{A}^{\mathbf{g}}) = f_Y(A^{\mathbf{g}}_{\tau}, H_{\tau}(\bar{A}^{\mathbf{g}}_{\tau-1}), U_Y)$$

Williams & Díaz (2021) have developed the lmtp package in R for estimating time-varying treatments with time-varying confounding. Among the many excellent features of their software is that it uses semi-parametric estimators, which can be specified from the Polley et al. (2023) library. Readers working with `lmtp` might find the following collection of tools useful for evaluating assumptions, creating graphical outputs and tables, and automating reporting: Bulbulia (2024).

References

Bulbulia, J. A. (2024). *Margot: MARGinal observational treatment-effects*. https://doi.org/10.5281/zenodo.10907724

Díaz, I., Williams, N., Hoffman, K. L., & Schenck, E. J. (2021). Non-parametric causal effects based on longitudinal modified treatment policies. *Journal of the American Statistical Association*. https://doi.org/10.1080/01621459.2021.1955691

Hoffman, K. L., Salazar-Barreto, D., Rudolph, K. E., & Díaz, I. (2023). *Introducing longitudinal modified treatment policies: A unified framework for studying complex exposures*. https://doi.org/10.48550/arXiv.2304.09460

Pearl, J. (2009). *Causality*. Cambridge University Press.

Polley, E., LeDell, E., Kennedy, C., & van der Laan, M. (2023). *SuperLearner: Super learner prediction*. https://github.com/ecpolley/SuperLearner

Richardson, T. S., & Robins, J. M. (2013). *Single world intervention graphs: A primer*. https://core.ac.uk/display/102673558

Robins, J. (1986). A new approach to causal inference in mortality studies with a sustained exposure period—application to control of the healthy worker survivor effect. *Mathematical Modelling*, *7*(9-12), 1393–1512.

Robins, J. M., & Richardson, T. S. (2010). Alternative graphical causal models and the identification of direct effects. *Causality and Psychopathology: Finding the Determinants of Disorders and Their Cures*, *84*, 103–158.

Williams, N. T., & Díaz, I. (2021). *lmtp: Non-parametric causal effects of feasible interventions based on modified treatment policies*. https://doi.org/10.5281/zenodo.3874931
